# Retraction: Overexpressed C14orf166 associates with disease progression and poor prognosis in non-small cell lung cancer

**DOI:** 10.1042/BSR-20180479_RET

**Published:** 2019-10-18

*Bioscience Reports* (2018) **38**(5); https://doi.org/10.1042/BSR20180479

The authors are retracting their published article as they have found that their conclusions linking C14orf166 expression to prognosis were not consistent with the Human Protein Atlas data (available at https://www.proteinatlas.org/ENSG00000087302-C14orf166/pathology/tissue/lung+cancer). This may be due to differences in sample ethnicity, and the small sample size used in their study. Due to this discrepancy, the authors believe that the results of the retracted article may not be representative of the Chinese lung cancer population.

The authors apologise for any inconvenience caused to the readers.

